# Supplementary material for: The interactome of intact mitochondria by cross-linking mass spectrometry provides evidence for coexisting respiratory supercomplexes
Source: Mol Cell Proteomics. 2017 Dec 8;17(2):216–32. doi: 10.1074/mcp.RA117.000470 (PMC5795388; doi:10.1074/mcp.RA117.000470)
Supplement: Supplemental Data [file supp_RA117.000470_133922_0_supp_23978_fzffwf.zip › spectra_annotation/mito_DR_spectra_annotation/84-1-2-1-6-1.pdf]

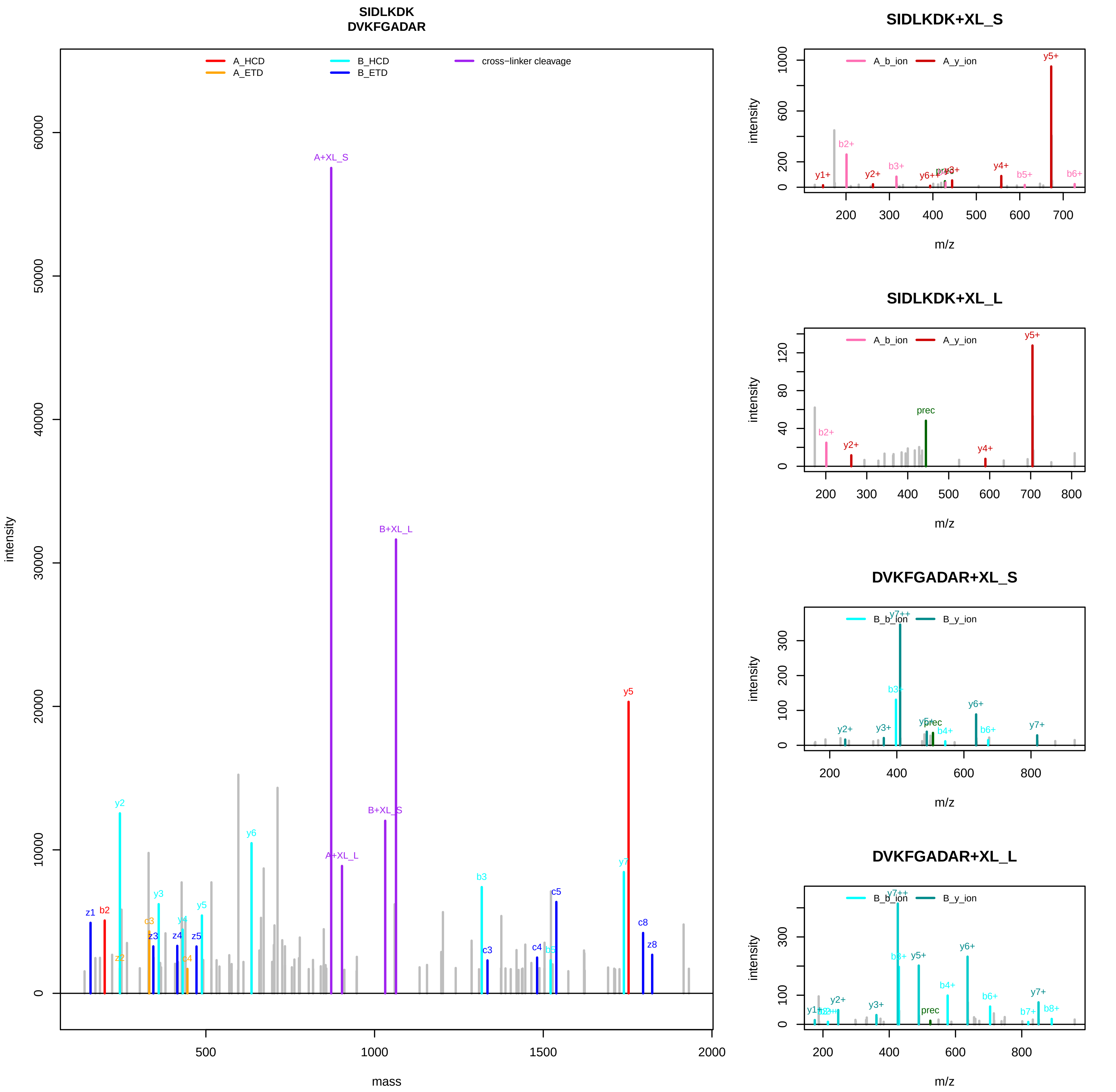
SIDLKDK
DVKFGADAR
A_HCD
A_ETD
B_HCD
B_ETD
cross−linker cleavage
A+XL_S
B+XL_L
B+XL_S
A+XL_L
y5
y7
b3
c3
c4
c5
c8
z8
y2
y3
y4
y5
y6
z1
b2
z2
c3
z3
z4
z5
c4
intensity
mass
SIDLKDK+XL_S
A_b_ion
A_y_ion
y5+
b2+
b3+
y1+
y2+
y6+
y4+
b5+
b6+
intensity
m/z
SIDLKDK+XL_L
A_b_ion
A_y_ion
y5+
prec
b2+
y2+
y4+
intensity
m/z
DVKFGADAR+XL_S
B_b_ion
B_y_ion
y7++
b3+
y6+
y2+
y3+
prec
b4+
b6+
y7+
intensity
m/z
DVKFGADAR+XL_L
B_b_ion
B_y_ion
y7++
b3+
y5+
y6+
b4+
y2+
y3+
prec
b6+
y7+
b7+
b8+
intensity
m/z
